# Segmenting or Summing the Parts? A Scoping Review of Male Suicide Research in Canada

**DOI:** 10.1177/07067437211000631

**Published:** 2021-03-15

**Authors:** John L. Oliffe, Mary T. Kelly, Gabriela Gonzalez Montaner, Paul S. Links, David Kealy, John S. Ogrodniczuk

**Affiliations:** 1 70439 School of Nursing, University of British Columbia, Vancouver, BC, Canada; 2 Department of Nursing, The University of Melbourne, Melbourne, Australia; 3 153002 Department of Psychiatry and Behavioural Neurosciences at McMaster University, Hamilton, ON, Canada; 4 175098 Department of Psychiatry, University of British Columbia, Vancouver, BC, Canada

**Keywords:** male suicide, men’s suicidality, men’s health inequities, adolescent male suicide, veterans mental health, suicide prevention

## Abstract

**Objective:**

Suicide in Canadian men is high and rising. Research consistently indicates increased suicide risk in male subgroups including sexual minority, Indigenous, middle-aged, and military men. The current scoping review addresses the research question: Among male subgroups featured in Canadian suicide research, what are the key findings to inform suicide prevention efforts?.

**Method:**

A scoping review was undertaken in accord with PRISMA-ScR guidelines. Structured searches were conducted in CIHAHL, Medline, PsychInfo, and Web of Science to identify studies reporting suicidality (suicidal ideation, plans and/or attempts) and suicide among men in Canada. Inclusion criteria comprised primary empirical studies featuring Canadian male subgroups published in English from 2009 to 2020 inclusive.

**Results:**

Sixty-eight articles met the inclusion criteria, highlighting significant rates of male suicidality and/or suicide in 3 categories: (1) health inequities (*n* = 29); (2) age-specific (*n* = 30); and (3) occupation (*n* = 9). The health inequities category included sexual minority men, Indigenous, and other marginalized males (i.e., homeless, immigrant men, and men who use opiates). Age-specific men focused on adolescents and youth, and middle-aged and older males. Active military, veterans, and first responders featured in the occupation category. Studies compared at risk male subgroups to females, general male populations, and/or other marginalized groups in emphasizing mental health disparities and increased suicide risk. Some men’s suboptimal connections to existing mental health care services were also highlighted.

**Conclusion:**

While male subgroups who are vulnerable to suicidality and suicide were consistently described, these insights have not translated to tailored upstream suicide prevention services for Canadian boys and men. There may be some important gains through integrating social and mental health care services for marginalized men, implementing school-based masculinity programs for adolescent males, orientating clinicians to the potential for men’s mid-life suicide risks (i.e., separation, bereavement, retirement) and lobbying employers to norm help-seeking among activate military, veterans, and first responder males.

## Abstract (fr)

**Objectif:**

Le suicide chez les hommes canadiens est élevé et en hausse. La recherche indique avec constance un risque de suicide accru chez les sous-groupes masculins, notamment la minorité sexuelle, les Autochtones, les hommes d’âge moyen et les militaires. Le présent examen de cadrage aborde la question de la recherche: chez les sous-groupes masculins qui figurent dans la recherche sur le suicide canadien, quels sont les principaux résultats pouvant éclairer les initiatives de prévention du suicide?

**Méthode:**

Un examen de cadrage a été entrepris conformément aux lignes directrices PRISMA. Des recherches structurées ont été menées dans CIHAHL, Medline, PsychInfo, et Web of Science afin d’identifier les études abordant la suicidabilité (idéation suicidaire, plans et/ou tentatives) et le suicide chez les hommes au Canada. Les critères d’inclusion comprenaient les principales études empiriques portant sur des sous-groupes masculins canadiens publiées en anglais de 2009 à 2020 inclusivement.

**Résultats:**

Soixante-huit articles satisfaisaient aux critères d’inclusion, démontrant des taux significatifs de suicidabilité masculine et/ou de suicide dans 3 catégories: 1) inégalités en santé (n = 29); 2) d’un âge spécifique (n = 30); et 3) emploi (n = 9). La catégorie des inégalités en santé comprenait les hommes de minorité sexuelle, autochtones, et autres hommes marginalisés (c.-à-d., itinérants, immigrants, et utilisateurs d’opiacés). Les hommes d’un âge spécifique se trouvaient chez les adolescents et les jeunes, et chez les hommes d’âge moyen et plus vieux. Les militaires actifs, les ex-militaires et les premiers intervenants appartenaient à la catégorie Emploi. Des études comparaient les sous-groupes masculins à risque avec les femmes, les populations générales masculines et/ou d’autres groupes marginalisés en mettant l’accent sur les disparités de santé mentale et le risque de suicide accru. Les liens sous-optimaux de certains hommes avec les services de santé mentale existants sont également présentés.

**Conclusion:**

Même si les sous-groupes masculins qui sont vulnérables à la suicidabilité et au suicide ont été systématiquement décrits, ces idées ne se sont pas traduites par des services de prévention du suicide sur mesure en amont pour les garçons et les hommes canadiens. D’importants progrès peuvent s’obtenir en intégrant les services sociaux et de santé mentale pour les hommes marginalisés, en instaurant des programmes de masculinité en milieu scolaire pour les adolescents, en dirigeant les cliniciens vers le potentiel de risques de suicide pour les hommes à mi-vie (c.-à-d., séparation, deuil, retraite) et en faisant pression auprès des employeurs pour qu’ils normalisent la recherche d’aide des militaires actifs, des ex-militaires et des hommes premiers intervenants.

## Introduction

In 2012, Canada passed the Federal Framework for Suicide Prevention Act, which instructed the federal government to consult with nonprofit organizations and relevant provincial and territory authorities to develop a suicide prevention framework.^1^ The Act acknowledged that suicide was a significant public health issue in Canada and that prevention was “everyone’s” responsibility (p. 1). The Act also required the Government to provide Canadians with an update every 2 years. In 2016, the Minister of Health responded with the first progress report, recommending collaboration between multilevel government and nonprofit organizations to address subgroups experiencing high rates of suicide, including immigrants, Indigenous peoples, federally incarcerated persons, and members of the Armed Forces. There was also a call to better disaggregate and disseminate population-based suicide data and evidence-based practices for prevention.^2^

In terms of tailoring to sub-populations, with the exception of maternal health, public health in Canada has remained gender neutral (i.e., vaccination, tobacco control, infectious disease control, etc.). This trend extends to suicide research and prevention. Despite consistent evidence that males die by suicide at 3 times the rate of females,^3^ boys and men have not featured as an “at risk” group nor have they been targeted with tailored suicide prevention efforts. Not only do males die by suicide at higher rates than females, from ages 10 through 60, men’s suicide rates increase in every decade of life, peaking in their 50s.^3^ Said plainly, through to their 6th decade, men become increasingly more likely to end their own lives, usually by violent means (firearms or asphyxiation by hanging). Although men in their 60s, 70s, and 80s die by suicide at lower rates compared to men 40 to 59 years old, sex differences prevail with Canadian male suicide rates > 60 years old being 19.3 versus 4.2 (per 100,000) for age-matched females.^3^

### Framing Male Suicide

Male suicide in Canada has been framed in numerous ways and diverse contexts. The gender paradox,^4^ for example, has long contrasted high male suicide rates with the more frequent suicide attempts in women.^5^ Men’s access to lethal means,^6^ complexities in their mental health help-seeking^7,8^ and substance use issues have been linked to their high and rising suicide rates in Canada.^9^ Layering onto these explanations have been assertions that, within the category of men, some male subgroups are comparatively more vulnerable to suicide.^10,11^ For example, higher suicidality (suicidal ideation, plans and/or attempts) and suicide risk among sexual minority men (SMM)^12^ and Indigenous men^13^ have been linked to health inequities. Male suicide has also been stratified and compared by age as well as by occupation to assign risk in lobbying for targeted prevention programs.^14,15^

Suicide research and prevention efforts are, however, scattered across numerous organizations and government levels in Canada.^2^ Although general population suicide crisis phone lines, chat services, and bereavement support groups exist across the provinces and territories,^16^ Canada operates without a designate suicide prevention research program focused on services for boys and men. With regard to upstream suicide prevention, the focus has been on better addressing mental illness in men.^17,18^ The most common mental illnesses implicated in male suicide are depression, anxiety, and substance use disorders.^19^ Herein, men’s mental health help-seeking and the fit of existing services are typically the focus.^20,21^ These help-seeking and service issues nonetheless require behavioral, social, and health system shifts (and synergies) to reduce male suicide.^22–24^ The current scoping review addresses the research question: Among male subgroups featured in Canadian suicide research, what are the key findings to inform suicide prevention efforts?

## Method

The current study was undertaken in accordance with the PRISMA reporting recommendations for scoping reviews.^25^

### Search Strategy

**Figure 1.**
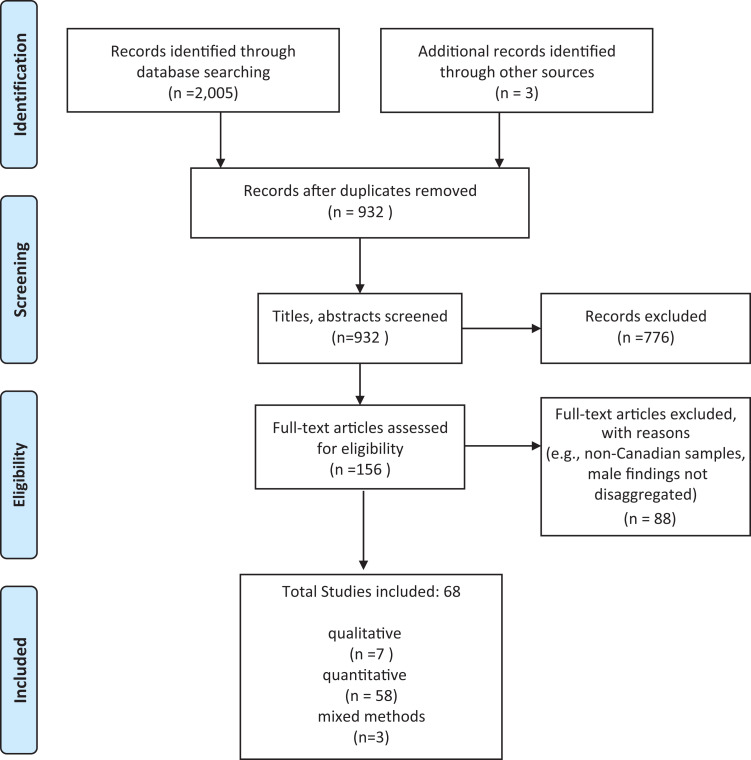
PRISMA flow chart.

A search was conducted in CIHAHL, Medline, PsychInfo, and Web of Science to identify research studies focused on suicide among Canadian boys and men. The inclusion criteria comprised primary empirical studies published in English from 2009 to 2020 inclusive. Three concepts were searched as subject headings and keywords in set combinations to produce a broad but focused retrieval of articles. The search concepts were suicid* (including “attempted,” “ideation,” “completed,” “psychosocial factors,” and “prevention”); “men” or “males” or “boys”; and geographic region, “Canada” and the province and territory names. Using Boolean operators to combine sets, these searches produced 1,096 articles. The articles were screened for inclusion by reviewing the title, abstract, and authors, and when necessary the article content. Articles were excluded if they did not focus on and/or report outcomes for a specific male subgroup; were conducted with non-Canadian samples; reviewed literature from non-Canadian samples; focused on neurological brain function or brain tissue autopsy; investigated mental health or self-harm or injury without a connection to suicidality; or pertained to medically assisted dying. After removing duplicates and articles that did not meet the inclusion criteria, 68 articles were retained for review (please see Figure 1—PRISMA flow chart).

The 68 articles included in the current scope were reviewed, and data were extracted and charted (please see Supplementary Table 1 Article Matrix). This process began by tabling author/year, study purpose, design, sample, and key findings for each study. Included were studies reporting predominately (85% to 90%) male samples and mixed sex research that disaggregated findings for men. In line with scoping review methodology,^25^ the reviewed articles were independently read by 3 researchers, compared, categorized, and allocated to subgroups based on the study sample and focus (please see Table 1 Articles by Category and Subgroup). These subgroups were the organizing principle to further read, review, and where possible compare the study findings in addressing the scoping review research question. Comparing male suicidality and suicide risk and protective factors across studies in each category, descriptive patterns were derived. To distil suicide prevention avenues, we drew from intervention study findings, author assertions regarding the applicability of their results, and/or our synthesis of the overall implications in making recommendations for Canadian male suicide prevention efforts.

**Table 1.** Articles by Category and Subgroup.

Category 1: Health inequities (*n* = 29)	
Sexual minority men (*n* = 12)	Qualitative: Ferlatte & Oliffe et al., 2019a; Ferlatte et al., 2019b; Ferlatte et al., 2019; Salway & Gesink, 2018 Quantitative: Ferlatte et al., 2018; Ferlatte et al., 2017; Ferlatte et al., 2015; Hottes, Ferlatte, & Gesink, 2015; Saewyc et al., 2020; Salway et al., 2018a; Salway et al., 2018b; Veale et al., 2017
First Nations/Indigenous men (*n* = 8)	Qualitative: Kral, 2016; Laliberte et al., 2009 Quantitative: Bombay et al., 2019; Fraser et al., 2015; Hajizadeh et al., 2019; Kumar et al., 2012 Mixed methods: Pollock et al., 2016; Tan et al., 2012
Marginalized men (*n* = 9) Immigrants (*n* = 3) Homeless (*n* = 3) Incarcerated (*n* = 2) Opioid dependent (*n* = 1)	Quantitative: Elamoshy & Feng, 2018; Marchand et al., 2017; Naud et al., 2010; Power & Ritchie, 2016; Saunders et al., 2017; Saunders et al., 2019; Sinyor et al., 2017; Torchalla et al., 2012 Mixed methods: Patterson & Holden, 2012
Category 2: Age-specific males (*n* = 30)	
Adolescents/youth (*n* = 21)	Quantitative: *n* = 21 *Studies of suicide* (*n* = 10) Dummer et al., 2010; Gontijo Guerra, 2016; Renaud et al., 2014; Rhodes et al., 2019; Rhodes et al., 2018; Rhodes et al., 2013; Rhodes et al., 2012; Sinyor et al., 2014; Skinner & McFaull, 2012; Soor et al., 2012 *Studies of suicidality* (*n* = 11) Conforti et al., 2020; Feng et al., 2016; Goodday et al., 2019; Goodday et al., 2020; Kim et al., 2019; Labelle et al., 2013; Langille et al., 2015; Newton et al., 2016; Peter & Roberts, 2010; Rhodes et al., 2014; Saewyc & Chen, 2013
Middle-aged and/or older men (*n* = 9)	Qualitative: Oliffe et al., 2011 Quantitative: Bardon et al., 2016; Burrows et al., 2011; Kisely et al., 2011; Ngui, Vasiliadis, & Préville, 2015; Sinyor, Schaffer, & Streiner, 2014; Vasiliadis, Gagné, & Préville, 2012; Zia et al., 2019 Mixed methods: Heisel et al., 2020
Category 3: Occupation (*n* = 9)	
Active military, veterans, and first responders (*n* = 9)	Quantitative: Afifi et al., 2106; Belik et al., 2009; Carleton et al., 2018; Mishara & Martin, 2012; Richardson et al., 2012; Rusu et al., 2016; Sareen et al., 2016; Sareen et al., 2017; Thompson et al., 2014

*Note*: *n* = 68.

## Results

The 68 articles were assigned to the following categories and subgroups: (1) health inequities (*n* = 29) comprising; (a) SMM (*n* = 12), (b) Indigenous men (*n* = 8), and (c) other marginalized men (*n* = 9); (2) age-specific males (*n* = 30), including; (a) adolescents and youth (*n* = 21; age range 9 to 25 years), (b) middle-aged and older males (*n* = 9; age range 35 to 54 years, 55 years and older); and (3) occupation (*n* = 9); (a) active military, veterans, and first responders (police, fire fighters, paramedics and emergency call dispatchers; *n* = 9).

### Health Inequities

Twenty-nine articles highlighted connections between men’s health inequities, suicidality and suicide making comparisons within subgroups and/or to general population samples.

#### SMM

The 12 articles focused on SMM comprised 8 quantitative^26–33^ and 4 qualitative studies.^34–37^ These articles pointed to high rates of suicidality and/or suicide within specific SMM subgroups, and/or in comparison to heterosexual males. Six cross-sectional survey studies reported gay and bisexual men’s (GBM) self-report of suicidality (suicidal ideation, plans and/or attempts).^26–28,30–33^ One survey (*n* = 8,382) indicated that half of the respondents experienced suicidal ideation (6 times higher than heterosexual men), with respondents who were experiencing 3 or more concurrent health problems (depression, anxiety, drug use, smoking, and HIV) being 16 times more likely to attempt suicide.^28^ A subsequent GBM survey (*n* = 7,995) emphasized that respondents living with HIV (*n* = 637) attempted suicide at 12 times the rate of men in the general population.^27^ Within-group GBM health inequities were also highlighted: Respondents with income less than $30,000 and without university education were 5 times more likely to attempt suicide.^26^ A transgender youth study (*n* = 923; 356 transmales) indicated that suicidal ideation among transmales was 5 to 8 times that of the general male population, with 65% of the respondents (14 to 18 years) having seriously considered suicide in the previous year.^32^

Diverse SMM suicidality risk factors were reported including social exclusion, lack of belonging, verbal and physical violence from others, homophobia, classism, and racism.^26,36^ Social and self-stigmas related to SMM status, HIV-positive status, and/or mental illness were also frequently reported predictors of suicidality.^27,31–37^ Ferlatte and colleagues^27^ reported that stigma and verbal abuse were associated with suicidal ideation, while social exclusion and physical violence were significantly associated with suicide attempts in GBM living with HIV. Stigma, in the form of discrimination or harassment, was strongly associated with suicide attempts in gay men.^31^

Experiences with mental health care services were connected to suicidality among GBM. In GBM (*n* = 1,480) who had experienced suicidal ideation or attempted suicide 58% (*n* = 858) had discussed a mental health concern with a health care provider (HCP), wherein 38% (*n* = 326) discussed suicide, 54% (*n* = 463) depression, and 17% (*n* = 146) substance use.^30^ Being open with HCPs about sexual identity, older age (> 50 years old) and having a large social network were positively associated with GBM’s engagement with mental health care.^30^ Contrasting this finding, gay men bereaved by suicide described how their deceased partner had refused professional help due to the stigma and shame associated with mental illness.^34^ To improve care for SMM, low-barrier, long-term, gender-affirming counseling and talk therapies were recommended,^35^ along with training for HCPs.^32,36^ Networks and peer support groups for GBM and school programs to reduce homophobia and stigma were suggested community-based strategies to improve social connection and reduce suicide risk.^30,33,35^

#### Indigenous men

The 8 studies reporting on Indigenous men included life histories,^38^ cross-sectional surveys,^39–41^ an historical–cultural review,^42^ suicide mortality data analyses,^43^ community-based consultations with elders,^44^ and an evaluation of crisis services.^45^ Study designs included 4 quantitative^38–41^ and 2 mixed methods.^44,45^ All study samples were mixed sex, and 6 explored suicide within Indigenous community settings. Four studies focused on Inuit people,^39,42,44,45^ 2 within First Nations communities,^38,43^ 1 addressed Metis people off-reserve,^41^ and 1 focused on off-reserve Indigenous groups.^40^

Studies revealed high suicidality and suicide rates among Indigenous males, relative to the general male population. High suicide rates among male Inuit youth 15 to 24 years (Nunavik, Quebec) were highlighted, as were lifetime suicide attempt rates, which were 10 times higher than males in the general population.^39^ Male Inuit living off-reserve reported the highest prevalence of suicidal ideation (22.7%) compared to off-reserve Inuit women and other First Nations groups.^40^ The prevalence of suicidal ideation among Metis people (*n* = 11,362) indicated male rates were higher (*n* = 3,079, 16.2%) compared to men who did not report Aboriginal identity (9.4%).^40^ Drawing from suicide mortality data, Indigenous males under 30 years of age accounted for 85% of suicide deaths in Labrador.^44^

In terms of suicide predictors, trauma and health inequities were frequently highlighted. Low-income and/or unemployment were associated with suicidality among Indigenous males.^40,41,43,44^ Male Inuit suicide rates were linked to colonialism, loss of culture, and government subjugation.^42^ Alcohol misuse and violence during adulthood were associated with suicide attempts for Inuit males (15 to 24 years).^39^ For Metis men, risk factors included major depressive episodes, history of self-injury, and divorce.^41,43^ Having a parent who was placed at an Indian Residential School was associated with suicidal ideation and attempts during adolescence and adulthood for First Nations males living on-reserve.^38^

Regarding mental health care services, Tan and colleagues^45^ reported that the majority of the calls to a Nunavut crisis line were distress-related (*n* = 2,858, 71.92%); males comprised 45% of callers (*n* = 1,800), though suicide concerns comprised only 8% of those calls. Health inequities comprising intertwined familial, systemic injuries including intergenerational trauma^38,44^ featured in lobbying income and employment assistance,^40^ and stronger efforts to empower communities to design and implement their own mental health promotion and suicide prevention programs.^42^

#### Other marginalized men

Among 9 articles, homeless (*n* = 3), immigrant (*n* = 3), incarcerated (*n* = 2), and opiate using (*n* = 1) men featured as marginalized groups with increased suicidality and/or suicide risk. Eight studies employed quantitative designs^46–53^ and one utilized mixed methods.^54^

Of the suicides in Toronto between 1998 and 2012 (*n* = 3,319), almost 9% (*n* = 290) were homeless or precariously housed persons.^52^ Of these 290—83% of the homeless and 75% of precariously housed—were men with a history of social isolation, interpersonal conflict, and marriage breakdowns.^52^ Drug and alcohol use (38%, *n* = 37), economic barriers (22%, *n* = 21), and family/relationship problems (18%, *n* = 17) were also linked to men’s homelessness wherein psychological pain was the strongest predictor of suicidal ideation.^54^ Childhood maltreatment was associated with increased suicide risk among homeless men (*n* = 489; 60% males, 40% Aboriginal).^53^

Recent male immigrants, compared to long-term immigrant residents, were reported to have lower rates of suicidal ideation^46^ and suicide^51^; however, the suicide risk among recent and long-term immigrant men was 3 times higher than immigrant females.^50^ Long-term male residents, those middle-aged or older and/or living in neighborhoods with low income levels had increased risk of suicide.^51^ Targeted suicide prevention strategies were recommended for established immigrants.^46^

Childhood physical abuse and neglect were significant predictors of male suicide attempts in a sex differences study of federally incarcerated adults (*n* = 415, males = 268).^49^ Naud and colleagues^48^ correctly predicted more than 60% of suicidality in male inmates (*n* = 1,047); however, among the men who died by suicide, 23 of the 26 did not express suicidality before their death.

Number of traumatic events was associated with lifetime suicidal ideation in long-term male opioid users.^47^ Conforming to masculine norms, including aggression and dominance, was connected with trauma histories for many male drug users.^47^

Across these marginalized men, suicidality was linked to social isolation (immigration, homelessness, incarceration experiences) compounded by complex personal histories comprising traumas,^47,49,53^ psychological despair,^54^ and lack of access to resources.^50–52,54^

### Category 2: Age-specific Males

#### Adolescents and youth

Of the 21 male adolescent and youth articles, 19 were epidemiological observational studies that drew from large population datasets or primary cross-sectional research.^55–73^ One study utilized mixed methods, combining data from the Quebec Coroner’s office and psychological autopsy interviews^74^ and one comprised a pre–post feasibility test of a literature based intervention.^75^

Ten studies compared suicide in male and female adolescents, linking coroner data or mortality statistics with provincial health databases.^55,57,65,66,68–74^ Study findings indicated male adolescent suicide rates being at least twice that of females.^66,72,73^ Males accounted for 70.2% of youth suicides in Toronto,^71^ 80% in Montreal,^74^ and 79% in Quebec.^57^ Rural Nova Scotia male youth suicide was reported to be 5.4 times higher than females.^55^ Compared to females, alcohol and drug use was higher in male youth suicides and less likely to involve a previous suicide attempt.^73^ Among 15- to 19-year-old males, suffocation rates increased annually, overtaking firearms as the leading suicide method.^72^

Health care use differed by sex among adolescents who died by suicide. Coroner and health data (*n* = 724; 532 = boys) indicated that compared to girls, deceased boys; (1) had fewer outpatient physician and emergency department (ED) visits, (2) were more likely to have no contact with health care, (3) were less likely to have contact in more than one health setting, and (4) were more likely to use the ED for nonmental health reasons.^68^ A health services study (*n* = 1,231 youth; *n* = 954 males) reported that 25% (*n* = 244) of males who died by suicide had not seen a HCP in the year prior to their death.^57^ Similarly, a case control study (*n* = 1657; 1,203 males) reported that only 35% (*n* = 99) of rural based males (*n* = 296) had accessed mental health services in the year prior to their suicide.^66^ Among deceased males, 18- to 25-years-olds were far less likely than 10- to 17-year-olds to have accessed the ED for mental health care.^66^ In a psychological autopsy study (*n* = 67 suicides; 52 male), men’s unmet health care needs were featured, underscoring the necessity for specialized care.^74^

Adolescent male suicide has been associated with depression, conflict with parents, and problems with romantic partners.^71^ Mood disorders and substance use were identified as contributing factors by family members of adolescents who died by suicide.^74^ A Nova Scotian study (*n* = 314,983 youth; 158,179 males) indicated that 12- to 24-year-old males from socially deprived and rural areas were most at risk for suicide.^55^

Ten studies explored suicidality in adolescents and youth.^56,58–64,67,70^ Sex differences studies indicated that suicide attempts in males occurred at a younger age (ages 11 to 13) compared to females (ages 14 to 16).^59^ A population study (*n* = 29,315; 48% males) reported that adolescent males were more likely than females to report physical violence (46% vs. 30%), and such experiences increased their odds 5-fold for making a suicide attempt; boys who experienced sexual violence were 3 times more likely than girls to make a suicide attempt.^70^ Boys who reported being bullied were 2 to 3.5 times more likely to report suicidal ideation, mental distress, and delinquency.^60^ Deviant behaviors were also a significant predictor of boys making a suicide attempt,^64^ and among high school students (*n* = 712; 360 males), boys with suicidal ideation scored higher on negative attribution style, hopelessness, and depression.^61^ Depression was associated with suicidal ideation and attempts among high school males (*n* = 9,225 students).^62^ Similarly, depressive symptoms in boys corresponded to a 1.75 times increased odds for suicidal ideation.^64^

In terms of protective factors against suicide attempts for male adolescents and youth, violence prevention,^70^ living in a two-parent family, and school connectedness were indicated.^62^ Evaluation of a literature based Cognitive Behavioral Therapy (CBT) curriculum indicated reduced suicidality in young males.^75^ Youth suicide related behaviors in Alberta EDs from 2002 to 2010 (*n* = 646,975) leveled, a finding attributed to the Alberta Suicide Prevention Strategy; however, boys were more likely than girls to present in the ED for self-cutting.^63^ In Ontario, ED presentations for suicidality after 2004 were 50% more severe events for boys, especially in regard to self-poisonings.^67^

#### Middle-age and older men

The 9 studies focused on middle age and older men highlighted specific life course factors associated with suicidality and suicide. Seven studies were quantitative designs,^24,76–81^ and 2 were qualitative.^82,83^ Canadian census and mortality data (*n* = 2,685,400) indicated that being older, unmarried, living alone, unemployed, low education, low income, and living in areas with higher social and material deprivation were most prominent in male suicide (*n* = 3,110).^77^ Older age, being single or widowed, having depression or anxiety, and using mental health services in the previous year were predictors of suicidal ideation in older men (>65 years; *n* = 2,494, 42% male).^80^ Of the 108 suicides in Nova Scotia in 2006, 90 (83%) were male with a mean age of 44 years old, the majority of whom were single.^78^ A case control study (*n* = 3,396 older adults) reported that older men (> 65 years) were 9 times more likely than women to die by suicide.^24^ The highest risk was in low education and high unemployment males who had accessed an ED, been hospitalized, or diagnosed with a mental health disorder.^24^ Among Toronto-based individuals who died by suicide (*n* = 2,886), the highest male suicide rates were in middle age (45 to 54 years old) and older men over 80 years of age.^79^ A study by Bardon and colleagues^76^ (*n* = 117; 60% male) reported that 26% of the people who died by suicide were isolated, and of these, 63% were males with no social or family contact amid experiencing mental health problems.

Regarding health services, middle-aged men who had died by suicide were more socially isolated and estranged from HCPs compared to previous decades.^76^ A cluster analysis of suicides in Toronto revealed middle-aged males with substance use problems, mental illness, and stressors such as criminal legal issues to have had little contact with psychiatry or ED in the week before death.^79^ In contrast, Kisely et al.^78^ reported that 75% of men who died by suicide in Nova Scotia had had some form of health service contact in the year preceding death, and in Quebec, older males who experienced suicidal ideation had accessed mental health care in the previous year.^80^ In terms of avenues for suicide prevention, Zia et al.^81^ study of men transitioning to retirement (*n* = 93; 54 to 78 years) indicated that brief screening tools may effectively identify middle-age and older men at risk of suicide. An evaluation of *meaning-centered men’s groups*, a 12-week program to reduce suicide risk and advance transitions to retirement through existential psychotherapy, reported increased psychological well-being and decreased depression, loneliness, and suicidal ideation amongst attendees (*n* = 30 men).^82^ A qualitative study of men 55 to 79 years old (*n* = 22) recommended community-based programs to enhance a sense of belonging and camaraderie for older men experiencing suicidal ideation.^83^

### Category 3: Occupation

#### Active military, veterans, and first responders

Nine articles reported on suicide risk amongst active military men (military men currently off work due to trauma were not delineated), veterans and first responders. Seven of these focused on suicidality among military personnel,^84–90^ 1 reported on suicidality among public safety personnel,^91^ and another included an evaluation of a suicide prevention program for Montreal police.^92^

Four military studies reported associations between occupational trauma and/or mental health disorders and the high rates of suicidality in military men.^85,87–89^ Military personnel (*n* = 6,700) reported significantly higher odds of lifetime and past year suicidal ideation and plans compared to the general population (*n* = 15,981).^88^ Regular Armed Forces personnel (*n* = 6,696; 86% male) had higher rates of past-year major depressive episodes, anxiety, and suicidal ideation compared with the general population (*n* = 25,113; 49% male).^87^ Depression severity was the most significant predictor of suicidal ideation among active Canadian Armed Forces (CAF), Royal Canadian Mounted Police (RCMP), and veterans seeking treatment (*n* = 250; 92% male).^86^ Significant increases in major depression, PTSD, anxiety,^87^ and lifetime suicide attempts were reported in military men with hazardous duty experience.^88^

Four studies attempted to disentangle military deployment trauma from lifetime trauma and/or psychiatric disorders to differentiate military-related suicidality.^84,85,87,88^ A history of being abused in childhood was higher in the Regular Armed Forces (47.7%) and Reserve Forces deployed to Afghanistan (49.4%) compared with the general population (33.1%), and this was associated with suicidality in CAF personnel (*n* = 8,161; 86% male).^84^ Rusu and colleauges^87^ suggested that military service may appeal to men with an increased risk of early onset mental health disorders, which may predispose them to work-related mental illnesses. Among active military personnel (*n* = 8,441), the number of interpersonal traumas experienced (i.e., sexual assault, child abuse) was directly associated with increased risk for suicide attempts.^85^ A study of suicidality in CAF (*n* = 8,161; 86% male) indicated most deployment-related traumatic events were significantly associated with suicidal behaviors.^89^ Veterans reporting 3 or more physical health conditions, and at least 1 mental health condition had a high prevalence of suicidal ideation (21.5%) compared to veterans with two or less physical and no mental health conditions (*n* = 2,658; 89% male).^90^ Compared to civilians, the use of mental health services was significantly higher among military who reported past-year suicidal ideation in both 2002 and 2012/13.^88^

A study of public safety personnel subgroups (*n* = 5,148; 66.6% male) including firefighters, police, and RCMP confirmed higher lifetime suicidality prevalence compared to the general population.^91^ Paramedics and correctional workers reported the highest prevalence of past-year and lifetime suicidality, with workers who were single, divorced, separated, or widowed more likely to report lifetime suicidality.^91^ In terms of suicide prevention, the evaluation of a program for Montreal police (*n* = 4,178; 77.9% male) indicated a 79% suicide rate decrease over 12 years.^92^

## Discussion

The findings from the current scoping review highlight the influence of health inequities, age, and occupation in delineating Canadian men’s suicidality and suicide. In addressing the research question, “Among male subgroups featured in Canadian suicide research, what are the key findings to inform suicide prevention efforts?”, it is clear that, in addition to mental illness diagnoses, a range of social and structural risk factors are consistently evident. For example, alcohol and substance use, bullying, child abuse, social isolation, employment barriers, low education, low income, divorce/relationship breakups, violence, and unmet healthcare needs permeate multiple male subgroups in diverse configurations to heighten men’s suicidality and suicide risk. Although predominately researched and reported in terms of discrete categories and subgroups, many men incur multiple inequities (e.g., Indigenous, Two-spirit, homeless) and live across several “at risk” subgroups (e.g., gay, middle aged, Veteran, retiree), which in combinations can serve to intensify men’s suicide risk. Considering such complexities, moving predominantly descriptive findings toward tailored male suicide prevention programs has proved challenging and demands integrated medical and social approaches.^93^

The body of knowledge (some of which is synthesized in the current scoping review) dedicated to nuancing *why* (i.e., inequities), *when* (i.e., adolescence and middle and older age), and *where* (i.e., military and first responders) many men experience suicidality and/or die by suicide has yielded few tailored prevention programs.^63,81,82,92^ It may be that disaggregating male suicidality and suicide by subgroups, while revealing important comparative and contextual information, has inadvertently stalled intervention efforts for reducing Canadian male suicide. In addressing the current article’s *Segmenting or Summing the Parts*? question, we offer the following three recommendations to transition the findings toward much needed and broadly inclusive men’s suicide prevention programs.

First, Canada’s lead and commitment to the social determinants of health—and by extension efforts for addressing health inequities—should be affirmed and directed toward tailoring men’s suicide prevention programs. Indigenous and SMM men have featured as experiencing significant disadvantages and disproportionality high suicidality and suicide. Related to this, it is estimated that only 25% of SMM who attempt suicide have accessed professional help,^30^ and it is also well-established that Indigenous men lack adequate mental health care services.^13^ There may be some benefits in programmatic efforts for reducing male suicide by building prevention services that serve ALL marginalized men. This is not to deny the unique and diverse traumas that marginalized men experience. Rather, this suggestion is made recognizing the intersections of marginality in many men’s lives and the budgetary constraints characterizing Canada’s public health care system. For instance, Two-Spirit Indigenous men experiencing homelessness, opiate use, and suicidality may endure societal and self-stigmas relating to any, and all of the afore listed locations. With skilled trauma-informed care inclusive of wide-ranging marginalized men, a gateway to connecting vulnerable men to diverse specialized services can be provided amid implementing safety plans to reduce their suicide risk. Pragmatically, such services may be more attainable (and sustainable) than the multiple asks requesting government support to start-up (and scale) an array of independent men’s suicide prevention services. Within these mental health care services, tangible connections to structural social supports in the form of housing and finance services would be especially key to supporting the mental health care of marginalized men.

Second, the work segmenting men’s suicidality and suicide by age also provides clues for optimizing the timing of prevention efforts. Findings from the current scoping review indicate that the gender paradox (wherein men, compared to women, are more likely to die by suicide than attempt suicide) begins in adolescence.^67^ Similarly, large-scale epidemiological youth suicide studies consistently highlight sex differences with fewer male mental health diagnoses and lower health care usage—issues that have been linked to violence in boys’ lives and the restrictive masculine codes socializing and governing boys actions (and immovability for help-seeking).^70^ Within this context, there could be significant gains for working with male adolescents and youth in schools to understand and proactively address some of the challenges experienced in their formative years.^75^ For example, boys can feel trapped inside confusing institutional narratives about being a good student and adult discourses of masculinity.^20^ School-based mental health promotion programs targeting male adolescents and youth are likely key to de-stigmatizing mental illness and norming help-seeking (and providing help to others) as strength-based manly actions.

The suicidality and suicide risks for middle-aged men consistently implicated relationship breakdowns, work- and finance-related stresses, and/or substance use.^76,77,79^ From a life course perspective, these (and other) psychosocial stresses can accrue over time to exhaust men’s efforts at countering, let alone enduring or recovering from such traumas.^18^ The transitional nature of common events (e.g., separation, bereavement, retirement) afford some important direction for orientating clinical and community services to significant challenges and changes experienced by middle-aged men. A community-based group work program with retired men, for example, revealed gains for meaning in life that bolstered resilience and reduced suicide risk.^82^ In addition to recommending government and industry investment in men’s community-based services, there is an imperative for upstream resources to recognize the mental health risks and harms that can accompany men’s midlife transitions.

Third, the current review underlines the need for employer provided mental health services for male military personnel, veterans, and first responders in Canada. In the context of military men, there is evidence that in addition to hazardous and trauma-related deployment, lifetime interpersonal traumas and adverse childhood experiences (ACEs) can predate military service to increase men’s suicidality risk.^84,85^ Male ACEs and military-related injuries invoke considerable shame and stoicism, and these trauma histories can be significant blind spots for treating clinicians. Similarly, the gendered dimensions of how male first responders can effectively work through occupational traumas warrants further investigation to norm mental health help-seeking as strength-based and men looking out for each other as a masculine value.^94^ More broadly, the workplace can be an ideal setting for male suicide prevention with in-service coaching about stress and anger management, relationship skills, and conflict resolution to increase the likelihood of men effectively self-managing and seeking additional help to prevent a crisis.^95^ Similar to the Montreal police program in the current review,^92^ workplace wellness efforts demand multiple components including prevention strategies to build social cohesion among men and reduce help-seeking stigmas.^94,96^ Specifically, industry-based training for all units, workers, supervisors and union representatives inclusive of crisis and safety plans with an emphasis on wellness can contribute to reducing workplace related male suicidality and suicide.^92,94,96^

The current scoping review includes limitations that might warrant systematic and/or meta-analyses to prioritize addressing specific variables in Canadian men’s suicidality and suicide prevention efforts. While the higher rates of Canadian male suicide reflect sex differences in many other countries,^97^ the specificities, contexts, and lack of tailored efforts for suicide prevention reported here cannot be generalized elsewhere. That said, our recommendations resonate with a 2020 Australian report by Poole,^98^ and benefits maybe garnered through cross-country research focused on male suicide prevention programs.^99^

## Conclusion

The current scoping review confirms male suicidality and suicide risks associated with health inequities, age, and occupation. Unfortunately, little traction has been made toward tailoring suicide prevention programs to target these male sub-groups nor systematically addressing men’s suicide in Canada more broadly. Almost a decade has passed since the 2012 federal framework named suicide a significant public health issue. Given the ongoing COVID pandemic has been shown to negatively impact the mental health of Canadian men, with 42% (*n* = 183) of help-seeking men in a recent study indicating suicidal ideation,^100^ it seems especially important to move quickly to advance interventions aimed at reducing male suicide.

## Supplemental Material

Supplemental Material, sj-docx-1-cpa-10.1177_07067437211000631 - Segmenting or Summing the Parts? A Scoping Review of Male Suicide Research in Canada: La segmentation ou la somme des parties? Un examen de cadrage de la recherche sur le suicide des hommes au Canada Click here for additional data file. Supplemental Material, sj-docx-1-cpa-10.1177_07067437211000631 for Segmenting or Summing the Parts? A Scoping Review of Male Suicide Research in Canada: La segmentation ou la somme des parties? Un examen de cadrage de la recherche sur le suicide des hommes au Canada by John L. Oliffe, Mary T. Kelly, Gabriela Gonzalez Montaner, Paul S. Links, David Kealy and John S. Ogrodniczuk in The Canadian Journal of Psychiatry

Supplemental Material, sj-pdf-1-cpa-10.1177_07067437211000631 - Segmenting or Summing the Parts? A Scoping Review of Male Suicide Research in Canada: La segmentation ou la somme des parties? Un examen de cadrage de la recherche sur le suicide des hommes au Canada Click here for additional data file. Supplemental Material, sj-pdf-1-cpa-10.1177_07067437211000631 for Segmenting or Summing the Parts? A Scoping Review of Male Suicide Research in Canada: La segmentation ou la somme des parties? Un examen de cadrage de la recherche sur le suicide des hommes au Canada by John L. Oliffe, Mary T. Kelly, Gabriela Gonzalez Montaner, Paul S. Links, David Kealy and John S. Ogrodniczuk in The Canadian Journal of Psychiatry
