# Supplementary material for: Segmenting or Summing the Parts? A Scoping Review of Male Suicide Research in Canada
Source: Can J Psychiatry. 2021 Mar 15;66(5):433–45. doi: 10.1177/07067437211000631 (PMC8107953; doi:10.1177/07067437211000631)
Supplement: Supplemental Material, sj-docx-1-cpa-10.1177_07067437211000631 - Segmenting or Summing the Parts? A Scoping Review of Male Suicide Research in Canada: La segmentation ou la somme des parties? Un examen de cadrage de la recherche sur le suicide des hommes au Canada [file sj-docx-1-cpa-10.1177_07067437211000631.docx]

Supplementary Table: Article Matrix (n=68)

|  |  |  |  |  |
| --- | --- | --- | --- | --- |
| **Author/year** | **Study Purpose** | **Design** | **Sample** | **Key Findings** |
| Afifi et al., 2016 | Examines child abuse exposure, SI, SP, and SA in CAF | Cross-sectional survey | n=24,142 adults 18-60 years | Child abuse exposure was higher in the Regular Forces (47.7%) and Afghanistan mission–deployed Reserve Forces (primarily male) (49.4%) compared with the general population (33.1%). Child abuse history had a more robust association with SI, SP and SA than deployment-related trauma. |
| Bardon et al., 2016 | Analysis and comparison of characteristics of persons who died by suicide in the Montreal metro transit system | Observation study | n=117 (2000-2008) 60% males  n=120 (1986-1995) 61% males | Five clusters of persons who died by suicide (n = 117; 2000-2008) included: Cluster 1: Isolated persons with multiple problems (26% cases), 63% male. Cluster 3: Men with a family and mood disorder (19% cases) 100% male. Cluster 4: Younger psychotic men (16% cases) 94% male. Recommended developing specific suicide prevention strategies based on characteristics of each cluster. |
| Belik et al., 2009 | Explores exposure to traumatic events and SAs among military personnel | Cross-sectional survey | n=8441 military personnel 16-54 years | Sexual and other interpersonal traumas (spousal abuse, child abuse) were significantly associated with SA. Exposure to combat or peacekeeping operations was not significantly associated with an increased risk of male SAs. |
| Bombay et al., 2019 | Explores parental IRS attendance and its association with SI and SAs in First Nations peoples | Cross-sectional survey | n=11,043 First Nations adults; n=4,837 First Nations youth | Having a parent who attended IRS was linked with increased risk for SI and SA in adolescent and adult cohorts. The link with SI in adults was significant only for males. |
| Burrows et al., 2011 | Examines the influence of social and material individual and area deprivation on suicide mortality | Longitudinal population cohort study | n=2,685,400 | Being male, older, unmarried, living alone, unemployed, less educated, low income, and living in areas with higher social and material deprivation associated with suicide. Recommended prevention strategies target males with low income who live alone in deprived areas. |
| Carleton et al., 2018 | Assesses SI, SP, and SA among Canadian public safety personnel | Cross-sectional survey | n=5,148; 66.6% male | Higher proportion of respondents reporting lifetime or past year SI, SP or SA than general population. Participants who reported being single, separated, divorced, or widowed were more likely to report lifetime SI, SA, and SA. Paramedics and correctional workers reported highest prevalence of past-year and lifetime suicidal behaviors. |
| Conforti et al., 2020 | Evaluates effects of literature-based CBT curriculum on suicidality in children | Prospective, pre-post test feasibility intervention | n=83 grade 7 and 8 children; 34% male, n=28 | For boys there was a 75% reduction in suicidality (ideation and behavior) scores approaching significance. Boys showed a 33% reduction in emotion dysregulation scores and 25% reduction on depression and anxiety scores. Children in high and low suicidality groups showed improvements. Recommended as a promising suicide prevention intervention. |
| Dummer et al., 2010 | Evaluates determinants of deaths in youth | Longitudinal population cohort study | n=314,983 youth 12-24 years; n=158,179 males; n=156,804 females | Suicides were 5.4 times higher and injury related deaths 2.8 times higher for males compared to females. Males were most at risk of death, especially those socially deprived and living in rural areas. Recommended prevention efforts target males in rural areas amid highlighting the need for additional data to identify socioeconomic and urban/rural trends. |
| Elamoshy & Feng, 2018 | Investigates effects of immigration identity on SI | Cross-sectional population-based survey | n=12,686 Canadians >15 years; n=1858 immigrants;  n=12,121 Canadians with SI, 44% male, n=5293 | Immigrants who had resided in Canada longer were more likely to report SI. Those with mood disorders 10 times more likely to report SI. |
| Feng et al., 2016 | Identifies high-risk neighbourhoods and other predictors of SI among children | Cross-sectional surveys | n=5,783 grade 5–8 students; 47.7% males, n=2,523 | 6.4% (n=340) of students indicated SI at least once in the previous 12 months. 13% of Aboriginal students reported SI, compared to 5.5% of non-Aboriginal students. No significant gender differences found. Recommended integrated parenting education, bullying prevention programs and mental health services for youth with depression, anxiety, low self-esteem. |
| Ferlatte et al., 2015 | Tests whether syndemics theory can explain suicidality in GBM | Cross-sectional survey | n=8,382 | GBM at a greater risk of SI than heterosexual men. SI and SA were positively associated with each GBM’s marginalization and psychosocial health problem. Each additional psychosocial problem increased prevalence of SI and SAs. |
| Ferlatte et al., 2017 | Evaluates how SI and SA among GBMSM-LWH are associated with stigma | Cross-sectional survey | n=673 | Elevated prevalence of SI and SA among GBMSM-LWH. Greatest association of SI and SA were observed when the cumulative effects of multiple forms of stigma were considered. |
| Ferlatte et al., 2018 | Describes variations in recent SA’s among GBM across socio-demographics | Cross-sectional survey | n=8,382 | Lower education and lower income interacted to increase the odds of recent SA. Decreased odd of recent SA for bisexual men in heterosexual relationships. Concluded that GBM (based on intersecting socio-demographics) are at unequal risk of recent SA. Recommended intervening with targeted services and programs. |
| Ferlatte, Oliffe, Salway, & Knight, 2019a | Explores the impact of gay men’s suicide on surviving male partners | Qualitative case study | n=2 | Suicide, mental illness, HIV, and sexual minority stigma were referenced as underpinning the refusal of gay men to seek professional help prior to suicide and these factors also intersected to produce complicated grief for bereaved male partners. Highlighted the need to de-stigmatize mental illness and suicide within and outside the gay communities. |
| Ferlatte, Oliffe, Louie, et al., 2019b | Informs programs and policies to understand and prevent suicide in GBTSM | Qualitative photovoice | n=29 | Four key findings in recommending; (1) recognizing and addressing enduring homophobia, biphobia, and mental illness stigma; (2) provision of low-barrier, long-term, and GBTSM-affirming counselling and talk therapy; (3) de-isolation through peer support and community connection; and (4) promoting creativity and cultural resilience. |
| Ferlatte, Oliffe, Salway, et al., 2019c | Explores the drivers of suicidality in GB2SM | Qualitative photovoice | n=21 | Three drivers of suicidality; (1) ACEs and negative adolescent experiences; (2) violence and homophobia disrupting men’s education and employment opportunities; and (3) social stigma and isolation. Recommended considering life-course trajectories and multiple social axes for targeted suicide prevention. |
| Fraser et al., 2015 | Assesses the prevalence of SI and SA among Inuit youth using a gender-based analysis | Cross-sectional survey | n=305 Inuit youth 15-24 years; 45% males, n=138 | Lifetime SI and SA higher among Inuit males of Nunavik compared to males throughout Canada. 22% males reported lifetime SA and physical violence and sexual abuse significantly associated with SA (controlling for alcohol use, marijuana use, and psychological distress). |
| Goodday et al., 2020 | Explores associations between exposure to maternal depression and other factors in childhood for subsequent youth SI and SA | Longitudinal survey | n=16,903 children and youth 0-25 years over 8 cycles 1994-2009 | Male youth exposed to maternal depressive symptoms as children did not show significantly higher rates of SI or SA. |
| Goodday et al., 2019 | Estimates the cumulative incidence of SI and SAs in male and female children and youth | Longitudinal survey | n=16,903 children and youth 11-25 years; 8 cycles of data collection; in final cycle n=13,169 youth, 6,568 males, 50% | Risk of SI and SA lower in males compared to females. SA occurred at younger age in males compared to females. Suggested findings had implications for SI and SA risk management by clinicians in recommending earlier implementation of suicide prevention programs. |
| Guerra & Vasiliadis, 2016 | Explores gender differences in healthcare service use prior to suicide | Descriptive statistics | n=1,231 youth ≤ 25 years; n=974 males; n=257 females | In the year before death, boys less likely than girls to have used healthcare services (74.9% vs. 82.5%), outpatient services (69.5% vs. 79.0%), been hospitalized (15.6% vs. 25.7%), and received a mental health-related diagnosis (33.1% vs. 46.7%). Recommended exploring nature and quality of care offered, building shared commitment between primary community mental health and specialty services. |
| Hajizadeh et al., 2019 | Assesses the socioeconomic inequalities in psychological distress and suicidality among Indigenous peoples | Cross-sectional survey | n=14,410 Indigenous Canadians off-reserve (Inuit, Metis, and First Nations); 45% males, n=6,510 | Income-related inequalities in suicidality higher among men. Inuit showed highest rates of SI (males=20.7%; females 24.5%) and SAs (males =1.6%; females 4.5%). Household income a protective factor against SI. Poorer individuals scored higher psychological distress, SI and SA’s. |
| Heisel et al., 2020 | Assesses the preliminary effectiveness of Meaning-Centered Men’s Groups (MCMG) for older men | Mixed methods | n=30 men ≥ 55 years | MCMG attendees reported significant increases for meaning in life, psychological well-being, life satisfaction, retirement satisfaction, general health, as well as decreases in depression, hopelessness, loneliness, and SI. Suggested upstream psychological interventions may serve an important role in mental health promotion and suicide prevention for older men. |
| Hottes et al., 2015 | Compares mortality rates for suicide and HIV in GBM | Observation study; national Death database, Stats Can | Used population attributable fraction data to derive and compare indirect estimates of mortality for HIV and suicide in GBM. | Suicide more significant cause of premature mortality among GBM than HIV. Burden of suicide estimated to be up to 3 times higher than HIV mortality. Suggested broadening the scope of GBM public health priorities to include suicide. |
| Kim et al., 2019 | Examines sex differences in cyberbullying, mental health, substance use, and SI among adolescents | Cross-sectional survey | n=4,940 students 43.3% male | Males exposed to traditional bullying were 2 to 3.5 times more likely to report SI, psychological distress, and delinquency. Compared to males never exposed, males with 2 or more exposes to cyberbullying reported increased risk of SI, psychological distress, and delinquency. |
| Kisely et al., 2011 | Explores health help-seeking prior to suicide in Nova Scotia | Descriptive statistics | n=108 suicides; 83% males, n=90 | Mean age at death was 44 years-old, majority were single. The 4^th^ decade of life had most suicide overall, and were the least likely to have suicidality documented in their medical records. |
| Kral, 2016 | Reviews risk factors and strategies for suicide intervention and prevention for Inuit | Narrative and historical review | Inuit comprise 5% of Indigenous population, ~50,000 in 2006 | Inuit are among highest suicide rates in the world, primarily male youth. Suicide epidemic must be viewed within the “government era”, between the 1950s and 1970s. Suicides began in 1980s and are increasing –10 times the rate of general Canadian population. Highlighted that community driven prevention programs led by Inuit have had some positive effects. |
| Kumar et al., 2012 | Explores the risk and protective factors associated with SI and SA among Metis adults | Cross-sectional survey | n=61,041 off-reserve First Nations; n=11,362 Metis | 13.3% of the 20-to-59-year-old Metis reported SI (46% male). Of these, 46.2% reported a lifetime SA. Prevalence of SI was higher among Metis men compared to men who did not report Aboriginal identity. Low income, non-thriving health, negative coping ability, history of foster care, major depressive episode, low self-esteem, and low social support correlated with SI among Metis men. |
| Labelle et al., 2013 | Examines cognitive variables, gender roles, and depression links to SI in adolescents | Cross-sectional survey | n=712 adolescents 14-18 years; n=360 males | Males reporting SI scored higher on negative attribution style, negative problem orientation, avoidant style, hopelessness, and depression (BDI-II). Negative problem orientation and avoidant style predicted SI in males. Prevention should be differentiated according to gender with emphasis on problem-solving skill building in males. |
| Laliberté & Tousignant, 2009 | Describes the psychosocial and behavioural characteristics of suicides among First Nations adults | Qualitative retrospective life histories/ psychological autopsies | n=30; median age 25 years; n=27 males | Suicide in this group over-represented by young single men. Alcohol intoxication at time of suicide, history of alcohol use (28/30) and drug use (23/30) were reported along with interpersonal and marital conflicts (22/30), violence (11/30), financial challenges (6/30) and social isolation (5/30). Recommendations for interventions with brothers of the deceased to reduce suicide clusters. |
| Langille et al., 2015 | Explores school connectedness, gender, and depression in suicidality among adolescents | Cross-sectional survey | n=4,365 grade 10-12 students; 50.8% males n=2,218 | School connectedness was a protective factor for males for SI (but not SA) when depression risk was included in the model. Higher SES scores were protective for SI and SA; living in a 2-parent family was protective for SA in males. Recommended increasing school connectedness as a universal adolescent mental health strategy. |
| Marchand et al., 2017 | Examines gender-specific factors associated with SI among people with opioid dependence | Cross-sectional survey | n=176; 54% male | Lifetime history of SI 43.8% (n =77) and of these 51.7% (n = 40) were male with average age of 19.82 years for first SI. For men, childhood emotional neglect and number of lifetime traumatic events were independently associated with lifetime SI. |
| Mishara & Martin, 2012 | Evaluates a suicide prevention program for the Montreal police | Mixed methods survey and interviews | n=4,178; 77.9% male | Suicide rate decreased by 79%, while other Quebec police rates had a nonsignificant (11%) increase. All members of the force were aware of and agreed with the importance of this resource.  Comprehensive suicide prevention programs tailored to the work environment may significantly impact suicide rates. |
| Naud & Daigle, 2010 | Examines the Suicide Probability Scale (SPS) within a male inmate population | Longitudinal survey | n=1,047 male inmates, mean age 33 years-old | The SPS measures correctly predicted more than 60% of SI and nonlethal self-injury cases, but 23 of the 26 completed suicides had not exhibited suicidality while incarcerated. Suggested adding complementary information to the SPS to more effectively screen inmates. Recommend ensuring follow-up care after release to reduce male suicide risk. |
| Newton et al., 2016 | Explores time trends in non-fatal ED suicidality related visits for adolescents | Population-based retrospective cohort study | n=646,975 adolescents 12-17 years | Fewer boys self-poisoned than girls; more boys self-cut than girls. Leveling of ED suicidality rates coincided with the Alberta Suicide Prevention Strategy introduced in 2006. Recommend ED visit rates as proxy for evaluating suicide prevention programs. |
| Ngui et al., 2015 | Determines the influence of individual and area-level characteristics associated with suicide in older adults in Quebec | Case–control design | n=3,396 adults ≥ 65 years (n=2,493 controls and n=903 suicides);  n=764 male controls and 79% male suicides, n=717 | At individual level, suicide was associated with male gender, age, presence of a physical and mental disorder and the use of health services. Risk of suicide among men was highest for those who had used the ED, been hospitalized and/or diagnosed with a mental disorder. |
| Oliffe et al., 2011 | Explores masculinity, depression and suicide among older men | Qualitative semi-structured interviews | n=22 men 55-79 years | SI related to depression secondary to cumulative losses around relationships, paid work and/or family deaths. Self-perceptions of failing to have built careers and/or wealth. Recommended clinicians ask older men about relationships and SI in directing this group to tailored virtual and community-based programs. |
| Patterson & Holden, 2012 | Assesses the theory of psychache in homeless men | Mixed methods survey and interviews | n=97 men; 19-77 years  24% (n=23) who had SA’s histories | Reasons given for homelessness included drug and alcohol use (38%), economics (22%), illness (20%), family/relationship problems (18%), lack of affordable housing (18%), and mental health difficulties (16%). Psychache, unbearable psychological pain, was reported to be a stronger predictor of SI than depression, hopelessness, or life meaning. |
| Peter & Roberts, 2010 | Determines sex differences between internalizing and externalizing influences on suicidality in adolescents | Longitudinal surveys | n=2,499 adolescents; 48.9% male | For males, depressive symptoms were associated with a 1.75 times higher likelihood of SI, and 1.25 times higher likelihood of anxiety. Deviant behavior was the only significant predictor of SA among males. Recommendations for gender-based modelling to understand suicidality in adolescents. |
| Pollock et al., 2017 | Explores suicide rooted in the historical, cultural, and socioeconomic context of Aboriginal communities | Population-based study | n=617 suicide deaths in Newfoundland, 84% males, n=522.  Aboriginal suicide deaths in Labrador n=128, 86% males, n= 110 | Males accounted for majority of suicides (~ 85%) with men < 30 years-old in Labrador Aboriginal communities most affected during period 1993-2009. Aboriginal communities in northern Labrador had the third lowest per capita income and highest suicide rates. Need for prevention programs to target high-risk sub-regions. |
| Power et al. 2016 | Examines childhood abuse, SA, and non-suicidal self-injury (NSSI) among incarcerated adults | Cross-sectional survey | n=415; 64% males, n=268 | Physical abuse and physical neglect were significant predictors of male NSSI and SA, respectively. Sexual and physical abuse were more strongly associated with SA than other types of abuse. |
| Renaud et al., 2014 | Determines health care services accessed in year prior to suicide | Mixed methods | n=67 suicides, ≤ 25 years-old; n=56 controls ≤ 25 years. 80% of suicides (and control group) were male | The suicide cohort were more likely than control group to have a psychiatric diagnosis, and need services to address substance use, depression and interpersonal distress. Only 40% of deceased accessed health services in the year preceding death. In the month preceding death, only 7.5% received second-line specialized mental health services. Most common unmet need in the suicide cohort was assessment and/or referral to another level of service, most often related to substance use and depression. |
| Rhodes et al., 2012 | Examines sex differences and potential misclassification of death among young suicide victims | Retrospective study | n=2,508 youth 10-25 years; n=1,294 suicides; n=254 undetermined deaths; n=961 accidental deaths | Males comprised 75% of suicide deaths and 62% were by asphyxia; majority were 16-25 years old. Over 50% lived with parents. Suicides by shooting were exclusive to males, mostly long guns. Sex differences in suicide rates were not due to misclassification. Need for more investigation of explanations for sex differences in youth suicide rates. |
| Rhodes et al., 2013 | Explores sex differences in health service use among young suicides | Retrospective study | n=724 children and youth 10-25 years; 73% males, n=532 | 80% had contact with health care system, and one-half presented to the ED in the year before death. Boys had fewer outpatient physician and ED contacts than girls. Boys less likely to have contact in more than one health setting. Among ED users, there was less use of the ED for mental health problems in boys compared with girls, and boys were more likely to use the ED for non-mental health problems. Recommended integration of mental health care across settings. |
| Rhodes et al., 2014 | Explores time trends in ED presentations for suicidality in adolescents following regulatory warnings for anti-depressants | Retrospective longitudinal study | n=2,502,017 adolescents 12-17 years; n=15,739 adolescents with ED related suicidality | Severity of ED presentation was greater overall after regulatory warnings and later economic recession. Decline in the proportion of boys  presenting to the ED due to “other methods” of SA (hanging and firearms), consistent with declining suicide rates in boys. Need for further research into the reasons for varying responses in boys and girls by SRB method. |
| Rhodes et al., 2018 | Explores medical care among youth suicides | Population-based case control study | n=1657 youth 10-25 years; 72% males, n=1203 | Older males (18-25) were less likely than younger males (10-17) to access the ED (ambulatory care only). The positive association with accessing ED mental health care (ambulatory care only) was almost 50% lower among older (18-25) males than younger (10-17) males, regardless of where they lived. Recommended prevention efforts address geographical and age-related barriers, intervening early and integrating services, including the ED. |
| Rhodes et al., 2019 | Investigates ED diagnoses and suicide among youth | Observational case-control study | n=1024 suicides 10-25 years; 68% males, n=697 | Male youth with a mood or psychotic disorder high-risk group for suicide. Schizophrenia/schizotypal/delusional disorders, mood disorders, and self-inflicted cut/pierce injury were associated with suicide in males. Suggestion that EDs can act as site for suicide prevention. |
| Richardson et al., 2012 | Examines SI in relation to PTSD, MDD, GAD, and AUD in veterans | Cross-sectional survey | n=250; 92% male | Self-reported depressive symptom severity was most significant predictor of SI. Majority met criteria for PTSD (73%) and 70% screened positive for MDD. PTSD symptoms associated with SI but not when depressive symptom severity and anxiety were controlled for. Important to assess for comorbidity, especially MDD, and SI when assessing veterans for PTSD. |
| Rusu et al., 2016 | Examines past-year mental disorders and suicidality in CAF | Cross-sectional survey | n=6,696 CAF, 86% male; n=25,113 general population, 49% male | Regular Force personnel had significantly higher rates of past-year MDE, GAD, and SI and past year SAs than general population. 45% of CAF sample had deployed to Afghanistan, and this sample had higher rates of MDE, PTSD, and GAD. Future research might examine occupational trauma, and determine why military service may appeal to males with an increased risk for early-onset mental illness. |
| Saewyc & Chen, 2013 | Examines adolescent SA and violence exposure | Cross-sectional population survey | n=29,315 adolescents 12-19 years; 48% male | Youth who had experienced any violence were 6 times more likely to attempt suicide. Two-thirds of boys reported experiencing at least one form of violence, verbal, physical, or sexual. Boys were more likely to report physical violence; physical violence increased the odds of SA nearly five times for boys (and girls). Violence prevention may be a primary strategy for suicide prevention. |
| Saewyc et al.,  2020 | Examines community and political links to suicidality in LGB adolescents. | Cross-sectional survey | n=2,678, 12-19 years. 46% male. LGB n=998, male 33% n=330. | In contrast to LGB females community level variables were not associated with suicidal behaviour in sexual minority adolescent males. Recommended clinicians, policymakers, and youth workers continue to advocate for LGBTQ friendly resources and inclusive community events. |
| Salway & Gesink, 2018 | Documents life stories of gay men who had SA as adults | Qualitative  narrative analysis | n=7 | Trauma and stress narratives enabled coping and acknowledged stigma and associated minority stress as cause of SI. Post-gay narratives warned of suicide among older gay men who feel erased from the gay movement in the contemporary culture of queer diversity. Pride narratives resisted connections to suicide. Findings suggest identity concealment remains a potential driver of gay men’s SAs. |
| Salway, Ferlatte, et al., 2018a | Describes rates of health care engagement among GBM with a history of SI or SA | Online survey | n=7,872 | Older age, larger social support networks, and being out to a health care provider about one’s sexuality were all positively associated with mental health care engagement. |
| Salway, Gesink, et al., 2018b | Estimates multiple mediating pathways in associations between stigma and SA among adult GBM | Online survey | n=7,872 | 3.4% reported SA attempt in the preceding 12 months. Multiple constructs of antigay stigma associated with SA; however, mediating pathways differed by construct, suggesting a combination of strategies is required to prevent suicide in GBM. |
| Sareen et al., 2016 | Compares trends in suicidality and the use of mental health services among Canadian military personnel | Comparative analyses | 2002 - n=5,153 military  2013 - n=6,700 military  2002 - n=25,643 general population  2012 - n=15,981 general population | In 2012/13 male military personnel had significantly higher odds of both lifetime and past-year SI and SP than the general population. Use of mental health services was significantly higher among military reporting SI compared to general population. Recommended occupations such as firefighting and law enforcement to employ screening and anti-stigma campaigns similar to those used by the military. |
| Sareen et al., 2017 | Examines DRTEs and past-year SI, SP, and SA | Cross-sectional survey | n=8,161 CAF 18-60 years | Lifetime deployment was not significantly associated with suicidality. Various DRTEs had different impacts; in models adjusting for mental disorders and child abuse, most DRTEs and number of types of DRTEs became nonsignificant in relation to all suicidality (SI, SP, and SA). |
| Saunders et al., 2017 | Describes trends in suicide and ED visits for self-harm in youth by recent immigrant status | Linked longitudinal population-based study | n=2.5-2.9 million per cohort period; 10-24 years | Suicide rates were highest among male long term residents. Male recent immigrants had higher suicide rates compared to females (2.7 to 7.2 per 100,000 versus 1.9 to 2.7 per 100,000). Male youth had almost 3 times greater risk of suicide compared with females. Recommended continued monitoring to facilitate early identification and targeted, culturally appropriate public health interventions for immigrant youth. |
| Saunders et al., 2019 | Estimates the rates of suicide and self-harm among recent immigrants to Ontario | Linked population-based study | n=590,289 recent immigrants; n=8,464,790 long-term residents | Suicide rates among recent immigrant men were 4.4/100,000 person-years versus 18.2/100,000 among long-term resident men (both higher than versus female cohorts). Suggested Canada’s immigration and selection policies targeting healthy educated individuals may offer suicide protection for immigrants. |
| Sinyor et al., 2014 | Explores bullying as a contributing factor to youth suicide | Observational study | n=94 children and youth 10-19 years | 70.2 suicide deaths were male with mean age of 16.8 years. Hanging and jumping from a height were the most common methods.  Bullying was present in 6 deaths (6.4%); Most common factors identified by the coroner were depression (51.3%) and conflict with parents (27.0%). |
| Sinyor et al., 2014 | Determines clusters based on factors related to suicide | Observational study | n=2,886 | Male deaths exceeded females at rate 2.5 to 3 three times across all ages. 5 clusters included varying degrees and configurations of depression, SA, resent stressors, marital status, mental illness, and substance use. |
| Sinyor et al., 2017 | Identifies factors associated with suicide in homeless and precariously housed people | Observational study | n=3,319 suicides; n=60 homeless, n=230 precariously housed | Homeless and precariously housed people are over-represented in suicide (9%) in a large urban center, and differ demographically, clinically, and in suicide method from non-homeless people who suicide. Homeless people were 83% male, more likely to have been seen at an ED or outpatient psychiatrist in the week prior to suicide. Recommended targeted suicide prevention strategies for homeless people. |
| Skinner & McFaull, 2012 | Longitudinal examination of suicide among children and adolescents | Retrospective longitudinal study | Children and adolescent (10-19 years) suicide data 1980-2008. | Suicide rates among boys aged 10–14 years showed no significant change during the 29-year span. Male suicides for 15-19 year-olds showed downward trend, but suicides by suffocation increased annually by an average of 1.8% for this age group. Among adolescents aged 15–19 years, overall suicide rate in 2008 accounted for 23% of all deaths (*n* = 208, 67% male); suffocation was the primary means. |
| Soor et al., 2012 | Identifies patterns of suicide among adolescents | Retrospective study | N=370 adolescents 11-18 years | Male adolescent suicide was twice as common as female suicide. Males were slightly older than females but occurred earlier (age 11-18). Males were more likely to use violent methods and less likely than females to have history of SA. Alcohol and drug involvement was more common in males. Psychiatric treatment most common factor identified in males (and females) but present in only 18% of sample. |
| Tan et al., 2012 | Assesses an Inuit crisis line and the demographic of the callers, call contents, and types of assistance provided | Mixed Methods | n=3,974 45% male, n=1,184 | Males made distress calls were less frequent than females. Males were more likely to make prank, abusive or wrong number calls than females. Distress calls focussed on relationships (26%), loneliness or boredom (17%) and suicidality (8%). Suggested the highest male suicide risk group (15-19 years) are not using the crisis line in recommending a line dedicated to young people in the north. |
| Thompson et al., 2014 | Explores physical and mental health problems in relation to SI among veterans | Computer-assisted telephone interview survey | n=2,658; 89% male | 81% of respondents had one or more diagnosed physical conditions, and 23% had one or more diagnosed mental health conditions. Almost all with mental health conditions had co-morbid physical conditions (95%), whereas only about a third with physical conditions had mental health conditions. SI was associated with gastro-intestinal disorders, depression, or anxiety and mood disorders. There was no direct association between SI and deployment. |
| Torchalla et al., 2012 | Explores childhood maltreatment subtypes and suicide risk among homeless adults | Cross-sectional survey | n=500 adults 19-66 years; 60% male | Majority of sample was at risk for suicide at the time of the assessment (70% had drug dependence issues). All subtypes of maltreatment, except physical neglect were related to increased suicide risk. The extent of child maltreatment was more significant than the specific subtype. |
| Vasiliadis et al., 2012 | Informs public health suicide prevention campaigns for older adults | Cross-sectional surveys | n=2,494 adults ≥ 65 years | In males, SI was associated with older age, being single or widowed, presence of depression or anxiety, and use of mental health services in the past year**.** Males with SI were less likely to be dispensed antidepressants than females. |
| Veale et al., 2017 | Documents prevalence of mental health problems among transgender youth | Cross-sectional survey | n=923 youth; n=216 trans men 19-25 years; n=140 trans boys 14-18 years | 75% of transgender boys and non-binary individuals 14-18 years reported self harm at least once in past year.  Transgender youth had higher risk of reporting psychological distress, self-harm, major depressive episodes, and suicide. 65% of transgender 14-18-year olds seriously considered suicide in the past year compared with 13% in the British Columbia Adolescent Health Survey. |
| Zia et al., 2020 | Investigates the psychometric properties of the SBQ-5 and GSIS-Screen among middle-aged and older men | Cross-sectional surveys | n=93 men 54-78 years | SBQ-5 was positively associated with the GSIS-Screen at the eligibility assessment. Only the GSIS-Screen uniquely predicted future depression and hopelessness ratings. GSIS-Screen was also significantly associated with feelings toward retirement. Suggested that brief screening tools may be effective in identifying SI in community-residing middle-aged and older men. |

ACE: Adverse childhood experience; AUD: Alcohol Use Disorder; BDI-II: Beck Depression Inventory II; CAF: Canadian Armed Forces; DRTE: Deployment-related traumatic events; ED: Emergency department ; GAD: Generalized Anxiety Disorder; GB2SM: Gay, bisexual and two-spirit men; GBM: Gay and bisexual men; GBMSM-LWH: Gay and bisexual men living with HIV; GBTSM: Gay, bisexual and two-spirit men; GLB: Gay, lesbian and bisexual; GSIS-Screen: Geriatric Suicide Ideation Scale-Screen; MDD: Major Depressive Disorder; MDE: Major depressive episode; PTSD: Post-traumatic Stress Disorder; SA: Suicide attempt; SBQ-5: Suicidal Behaviors Questionnaire; SI: Suicide ideation; SP: Suicide plan; SRB: Suicide related behavior
